# Extracellular Lipase and Protease Production from a Model Drinking Water Bacterial Community Is Functionally Robust to Absence of Individual Members

**DOI:** 10.1371/journal.pone.0143617

**Published:** 2015-11-23

**Authors:** Graham G. Willsey, Matthew J. Wargo

**Affiliations:** Department of Microbiology and Molecular Genetics, University of Vermont College of Medicine, Burlington, Vermont, United States of America; Argonne National Lab, UNITED STATES

## Abstract

Bacteria secrete enzymes into the extracellular space to hydrolyze macromolecules into constituents that can be imported for microbial nutrition. In bacterial communities, these enzymes and their resultant products can be modeled as community property. Our goal was to investigate the impact of individual community member absence on the resulting community production of exoenzymes (extracellular enzymes) involved in lipid and protein hydrolysis. Our model community contained nine bacteria isolated from the potable water system of the International Space Station. Bacteria were grown in static conditions individually, all together, or in all combinations of eight species and exoproduct production was measured by colorimetric or fluorometric reagents to assess short chain and long chain lipases, choline-specific phospholipases C, and proteases. The exoenzyme production of each species grown alone varied widely, however, the enzyme activity levels of the mixed communities were functionally robust to absence of any single species, with the exception of phospholipase C production in one community. For phospholipase C, absence of *Chryseobacterium gleum* led to increased choline-specific phospholipase C production, correlated with increased growth of *Burkholderia cepacia* and *Sphingomonas sanguinis*. Because each individual species produced different enzyme activity levels in isolation, we calculated an expected activity value for each bacterial mixture using input levels or known final composition. This analysis suggested that robustness of each exoenzyme activity is not solely mediated by community composition, but possibly influenced by bacterial communication, which is known to regulate such pathways in many bacteria. We conclude that in this simplified model of a drinking water bacterial community, community structure imposes constraints on production and/or secretion of exoenzymes to generate a level appropriate to exploit a given nutrient environment.

## Introduction

In oligotrophic environments, particularly aquatic systems, acquisition of nutrients from complex biomolecules is one of the keys to success [1–3]. This acquisition is achieved through secretion of hydrolytic enzymes to generate soluble products that can be imported for metabolism within the cell [2]. Key to regulation of this behavior is the fact that enzymes carry a metabolic cost and that the soluble products of such enzymes are considered community property, being available to producers and non-producers alike [4, 5]. To avoid expending energy on un-recoverable enzymes and limit free-loading cheater cells, single species and mixed communities employ chemical signals to coordinate timing and extent of extracellular enzyme production [4, 6, 7]. Contribution of chemical signaling and community members to extracellular enzyme production has been studied extensively in single and dual-species cultures [for mixed species, see [8–11], among others], but the impact of individual species on the production of extracellular enzymes from more complex communities has received less attention.

In addition to in vitro studies, extracellular enzymes have been analyzed by direct sampling of enzymes produced by natural microbial assemblages in diverse environments. These studies focus primarily on the association of the extracellular enzymes with the nutrient conditions at the study site or in experimental microcosms [[12–15], among many others]. In these cases, the natural assemblage is intact and interspecies microbial communication is occurring. While important correlations between taxonomic groups and enzyme activities can be made, the complexity of the community renders microbiological dissection of the contribution of individual members difficult.

Exoenzyme production is often feedback controlled by the regulation of secreted hydrolytic enzymes proportional to acquisition of products [16]. Feedback regulation and chemical signaling mechanisms likely combine to dictate the overall production of hydrolytic enzymes within microbial communities. These regulatory processes would be predicted to result in exoenzyme production that is resilient to changes in the community under a given set of environmental and nutrient conditions. Complex biological communities often exhibit robustness to perturbation [[17] includes a review of robustness for microbial populations]. Robustness is the maintenance of function and resilience to change when exposed to changing conditions or changing species composition [18]. Functional stability is therefore often correlated with diversity in natural communities [19, 20]. This prediction has been experimentally demonstrated for increasing diversity [21] and species evenness [22]. The latter work, in particular, examined ecosystem function of a mixed community of denitrifiers and demonstrated functional robustness linked to initial species evenness [22].

We were interested in the concept of functional robustness in relation to extracellular lipase and protease production in a model drinking water microbial community. Secreted lipases and proteases are ecologically and medically important and have not been as well studied as secreted glycosidases, glucosidases, and amidases. In this study we have used members of a drinking-water bacterial community derived from the International Space Station Potable Water Delivery System [23] to systematically investigate the contribution of each community member on extracellular enzyme production by these model mixed-species communities. We determined that community production of most extracellular enzyme activities was robust to absence of any single species.

## Results and Discussion

### Characterization of the model mixed bacterial community

Our primary goal in this study was to assess exoenzyme production from our model mixed-species bacterial community. We chose cultivable isolates from the ISS potable water system [23] that were well-described, common, and abundant members of most drinking water biofilm communities [24, 25]. Members of drinking water and other biofilm communities often have distinct interactions that lead to stable association and community formation [26]. Thus, the isolation of the bacteria in this study from the same biofilm sample likely contributed to our success in establishing a reproducible model community.

Using a static culture system, the mixed-species bacterial communities form a mixture of robust biofilms and planktonic cells by 72 hours, which is the duration of the experiments reported below. We chose 72 h because all exoenzyme activities were highest at this point (data not shown) and were undetectable before 48 h. We acknowledge that a static growth model does not mimic the typical growth conditions for drinking water bacteria, but it was a necessary experimental artifice to allow accumulation and subsequent measurement of exoenzymes. Future work will examine biofilm formation by this community in a capillary flow cell system, similar to that described recently for another multi-species biofilm [27].

We initiated the communities with all species at the same optical density within the well, rather than the same number of cells (CFU/ml for 0.05 OD_600_ is reported in the Materials and Methods Section). We chose an approximately even initial community, as evenness has been shown to favor functional robustness in multispecies bacterial communities [22]. We also assessed the endpoint population proportions to better characterize this biofilm community. We exploited differential antibiotic sensitivities at different temperatures (see Table 1), which, coupled to colony morphology and colony color, enabled enumeration of all species. The proportions of each species in the complete community mix are shown in Table 2. Using basic community ecological analyses, the endpoint community in the presence of all species has a Simpson’s Index of 0.34, a *ln*-based Shannon Index of 1.92, and a Equitability Index of 0.87.

**Table 1:** Differential susceptibility of International Space Station isolates ^a^.

Species	T	Cb	Ct	Tp	Gm	Km
*Ralstonia pickettii*	37	++	-	+	-	-
	30	++	-	-	+	-
*Cupriavidus metallidurans*	37	-	-	-	-	++
	30	+	-	-	++	++
*Chryseobacterium gleum*	37	-	-	-	-	++
	30	-	-	+	++	++
*Ralstonia insidiosa*	37	++	-	-	++	+
	30	++	-	-	+	+
*Sphingomonas sanguinis*	37	-	-	++	-	-
	30	++	-	+	-	-
*Burkholderia multivorans*	37	++	++	-	+	+
	30	++	++	-	++	+
*Phyllobacterium myrsianacearum*	37 ^b^	-	-	-	-	-
	30	-	-	++	-	-
*Sphingomonas paucimobilis*	37	++	-	++	-	-
	30	++	-	++	-	-
*Burkholderia cepacia*	37	+	-	++	+	-
	30	-	-	+	++	-

Abbreviations: temperature °C (T); carbenicillin 100 μg/ml (Cb); cetrimide 200 μg/ml (Ct); trimethoprim 100 μg/ml (Tp); gentamicin 25 μg/ml (Gm); kanamycin 100 μg/ml (Km); normal growth compared to no selection (++); slow growth, less than half of colony size from no selection (+); no apparent growth (-).

^a^ Growth after 48 hours on LB medium supplemented with the listed antibiotics.

^b^ *P*. *myrsianacearum* grows exceedingly slowly in the absence of selection at 37°C.

**Table 2:** Biofilm percent composition at 72 h based on differential plating.

Species	+ *C*. *gleum*	- *C*. *gleum*	Significance ^a^
*Ralstonia pickettii*	5.9 (0.9) ^b^	4.5 (0.3)	not significant
*Cupriavidus metallidurans*	1.0 (0.5)	1.3 (0.8)	not significant
*Chryseobacterium gleum*	27.7 (2.1)	0	*** ^c^
*Ralstonia insidiosa*	18.7 (2.8)	19.6 (4.2)	not significant
*Sphingomonas sanguinis*	4.4 (1.5)	7.4 (0.8)	*
*Burkholderia multivorans*	17.7 (1.4)	21.9 (2.2)	not significant
*Phyllobacterium myrsianacearum*	4.0 (4.0)	1.4 (0.2)	not significant
*Sphingomonas paucimobilis*	9.3 (2.3)	15.7 (2.2)	*
*Burkholderia cepacia*	11.1 (1.7)	28.1 (2.1)	***

^a^ Statistical significance measured using two-way ANOVA with Sidak’s multiple comparison test.

* = p < 0.05

*** = p < 0.001

^b^ Percent composition shown with standard deviation in parentheses.

^c^ While the change in *C*. *gleum* composition is statistically significant, it is reported for completeness, since that was the experimental variable between these two groups.

This nine membered model bacterial community is relatively simple compared to many naturally occurring communities, although it is important to note that some natural extreme systems have fewer species [28]. Our goal in establishing a community with less than ten members was to incorporate sufficient diversity to give rise to abundant interactions but limit the possible permutations to an experimentally tractable quantity. The latter property is critical to dissect the molecular mechanisms governing enzyme production in the community. Using Reed’s Law of community interaction [29], the number of unique interaction combinations in a nine-membered community is 502. If we limit this to two and three-membered interactions, a compromise between the maximum of Reed’s Law and the strictly pair-wise assumption of Metcalf’s Law, there are still a possible 120 unique interaction sets within our artificial community. While we appreciate this is radically simplified compared to natural systems, it provides sufficiently complex interactions to function as a tool for reductionist approaches.

### Impact of community membership on lipase and protease activity

Bacterial exoenzymes are important contributors to ecosystem function [3] and secreted glycosidases, glucosidases, and amidases have been particularly well-studied ([30, 31], among many others). Lipases play important roles in acquisition of nutrients, particularly in waste water systems and food processing [9, 32, 33], but have been relatively understudied in relation to community composition. To understand the contribution of individual community members to lipase production, we measured extracellular enzyme activity for short chain lipases (ρ-nitrophenylbutyrate, NPB), long chain lipases (ρ-nitrophenylpalmitate, NPP), and phosphatidylcholine-specific phospholipases C (ρ-nitrophenylphosphorylcholine, NPPC) from community supernatants collected after 72 hours under static culture conditions. We measured the enzyme activities for each individual species alone, the complete community of all nine species, and multispecies collections each with one member absent from the community. It is important to note that while phosphatidylcholine-specific phospholipase Cs have the highest specificity for this substrate, other enzymes including phosphatases and esterases can hydrolyze NPPC [34]. Therefore, we have endeavored to describe on NPPC hydrolysis activity, regardless of source.

**Fig 1:**
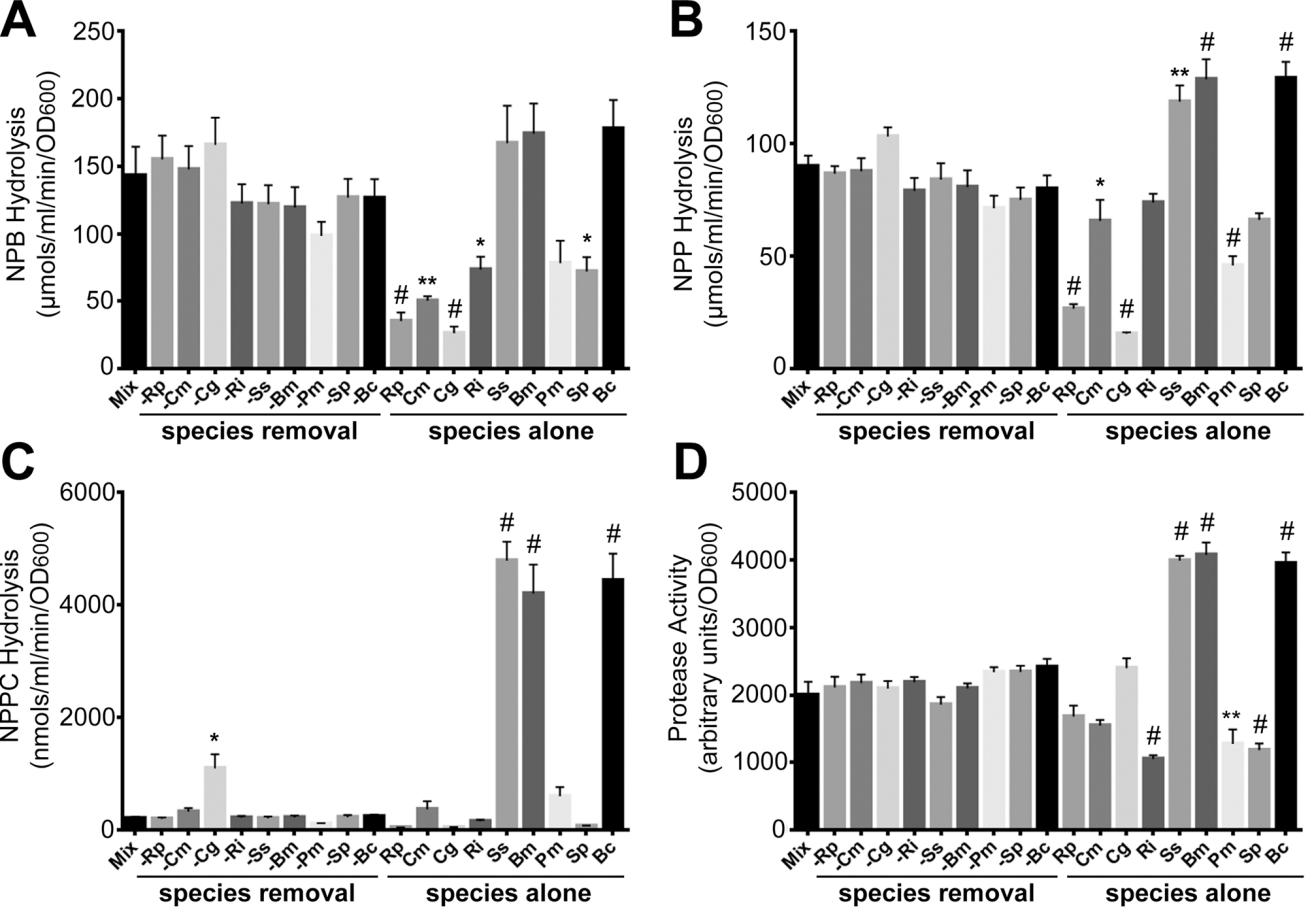
Effect of species absence on exoenzyme production from model communities. Static bacterial assemblages (‘Mix’ and ‘species absence’, where a minus symbol in front of the species abbreviation denotes removal) or single species cultures (‘species alone’) were assayed for (A) short-chain lipase activity using NPB, (B) long-chain lipase activity using NPP, (C) choline-specific phospholipase C activity using NPPC, and (D) protease activity using FITC-casein. Results are compiled from three separate experiments each with three biological replicates and error bars represent SEM. Samples all compared to ‘Mix’ (first bar) using ANOVA with Dunnett’s post-test. Statistical symbols: * = p < 0.05; ** = p < 0.01; # = p <0.001. Abbreviations: *Ralstonia pickettii* (Rp), *Cupriavidus metallidurans* (Cm), *Chryseobacterium gleum* (Cg), *Ralstonia insidiosa* (Ri), *Sphingomonas sanguinis* (Ss), *Burkholderia multivorans* (Bm), *Phyllobacterium myrsianacearum* (Pm), *Sphingomonas paucimobilis* (Sp), and *Burkholderia cepacia* (Bc).

Overall enzyme production for both short and long chain lipases was robust to absence of individual species, as demonstrated by the lack of statistically significant changes to NPB and NPP hydrolysis activity when any one species was left out of the community (Fig 1A and 1B, left side of each graph). However, it was obvious that each species alone produced varying enzyme activities (Fig 1A and 1B, right side of each graph). We observed functional robustness in NPPC activity for most communities, however, absence of *Chryseobacterium gleum* caused a substantial and significant increase in the resultant NPPC hydrolysis activity of the community (Fig 1C). We examined a potential mechanism for this change in hydrolysis activity in Section 3.5.

Like lipases, proteases have also been understudied from an ecological context in aquatic ecosystems. Using the same strategy as for the three lipid hydrolysis activities, we observed overall robustness of protease activity to absence of single species from these communities (Fig 1D, left side of the graph) compared to the variation in activity from each species grown alone (Fig 1D, right side of graph).

### Liposome stability in community supernatants

**Fig 2:**
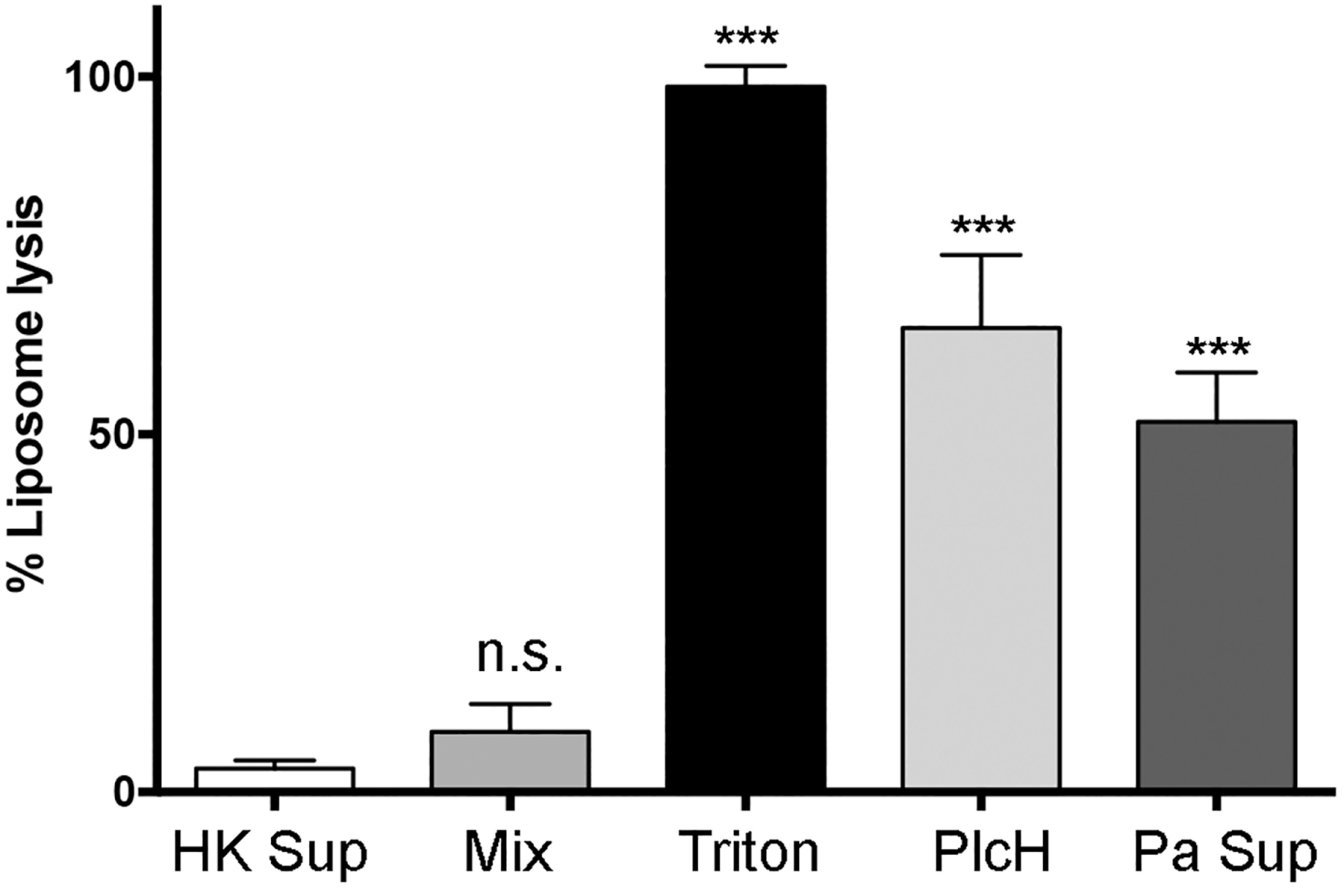
Liposomes were not significantly lysed by exposure to community exoproducts for four hours. This data is compiled from three independent experiments each with three biological replicates and error bars represent SD. Data was analyzed by one-way ANOVA and a Dunnett’s post-test was used to compare all groups to heat-killed biofilm supernatant ‘HK sup’ (first bar). Statistical symbols: NS = not significant; *** = p < 0.001. Abbreviations: Mix = supernatant from mixed culture; Triton = Triton X-100 treatment; PlcH = treatment with 1 ng/ul purified PlcHR (CITE); Pa Sup = supernatant from Pseudomonas aeruginosa PAO1 as a postitive lipolytic control.

One of our future goals is to study liposome interaction with this biofilm community, however, liposomes can also play a useful surrogate role as a model cell to study bacterial interactions with cell membranes. The above enzymatic activities were based on hydrolysis of dissolved substrates, whereas liposomes are more applicable as a potential cell-like target of such enzymes. We used a liposome formulation that mimics eukaryotic cells (phosphatidylcholine-rich) and measured the ability of secreted products to lyse these liposomes. We could measure no statistically significant liposome lysis by the culture supernatants compared to heat-denatured supernatant with liposomes as the negative control and Triton X-100 treatment as the positive control (Fig 2). We allowed these incubations to proceed for four hours, which was a sufficient time to achieve saturation for all colorimetric reactions. Beyond this time we did note destabilization of the liposomes, but this happened in the heat-denatured control as well, suggesting there may be non-enzymatic components in the community supernatants.

### Implications of lipase and protease robustness for microbial ecology

Many studies support the importance of species diversity in the establishment and stability of ecosystems [reviewed in [35, 36]] and functional biodiversity in ecosystem reliability [21]. Additionally, species evenness has been demonstrated to contribute to community dynamics, response to perturbations, and ecosystem functions [22, 37, 38]. Our study was not designed to directly address these concepts and our method of community establishment was based on the strategy from a single study on community evenness [22]. Given those caveats, our data generally support the extension of initial community evenness to extracellular lipase and protease production. However, it is important to note, as with other studies on microbial evenness [22], that the final community was much less even than the initial community (Table 2).

This study provides a partial examination of the effect of selective stress on a community; that is, a stress that specifically removes one or more species without being stressful to the rest of the community [39]. Specific examples in the natural world would be salt and desiccation stress [40], species specific predation [41], phage infection [42], or antibiotic treatment [43]. Here, absence of a single species would mimic selective stress in terms of removal of live organism, but does not mimic the consequences of in situ death due to such stresses, including released nutrients and altered biofilm structure.

Our study focused on lipases and proteases, therefore our data is particularly influenced by organisms producing the highest abundance of these products. In our community, it is important to note that three species (*B*. *cepacia*, *B*. *multivorans*, and *S*. *sanguinis)* could produce high levels of all the measured enzymes (Fig 1). Thus, removal of one of the three may not be expected to dramatically alter total production. Functional redundancy in microbial (and other) communities relates to robustness and has been termed the “insurance hypothesis” [44], such that a community will always have the capability to express a critical function. Future studies will examine the contribution of these three species to extracellular enzyme activities and will allow us to directly test whether functional redundancy between these lipase producers is responsible for the apparent robustness to species removal.

The above paragraphs describe the ecological underpinnings and consequences of robustness without specifically addressing the molecular mechanisms. We have not examined these mechanisms experimentally, but mechanism will be a target of future studies. We predict that robustness of lipase and protease production is likely due to the mixed community having multiple control mechanisms to optimize resource acquisition [16, 45]. While we have not directly examined the production of autoinducers or induction of quorum sensing in this bacterial community, all isolates are from species that commonly encode quorum sensing systems (via searches of JCVI and SMART databases). Based on previous studies of dual species mixtures, it is likely that both intraspecies and interspecies signaling will be taking place in this model system [8, 11, 46, 47] and signal interference may play a key role in sculpting the final regulation of community property [48, 49].

### Examination of the *C*. *gleum* effect on NPPC hydrolysis activity

**Fig 3:**
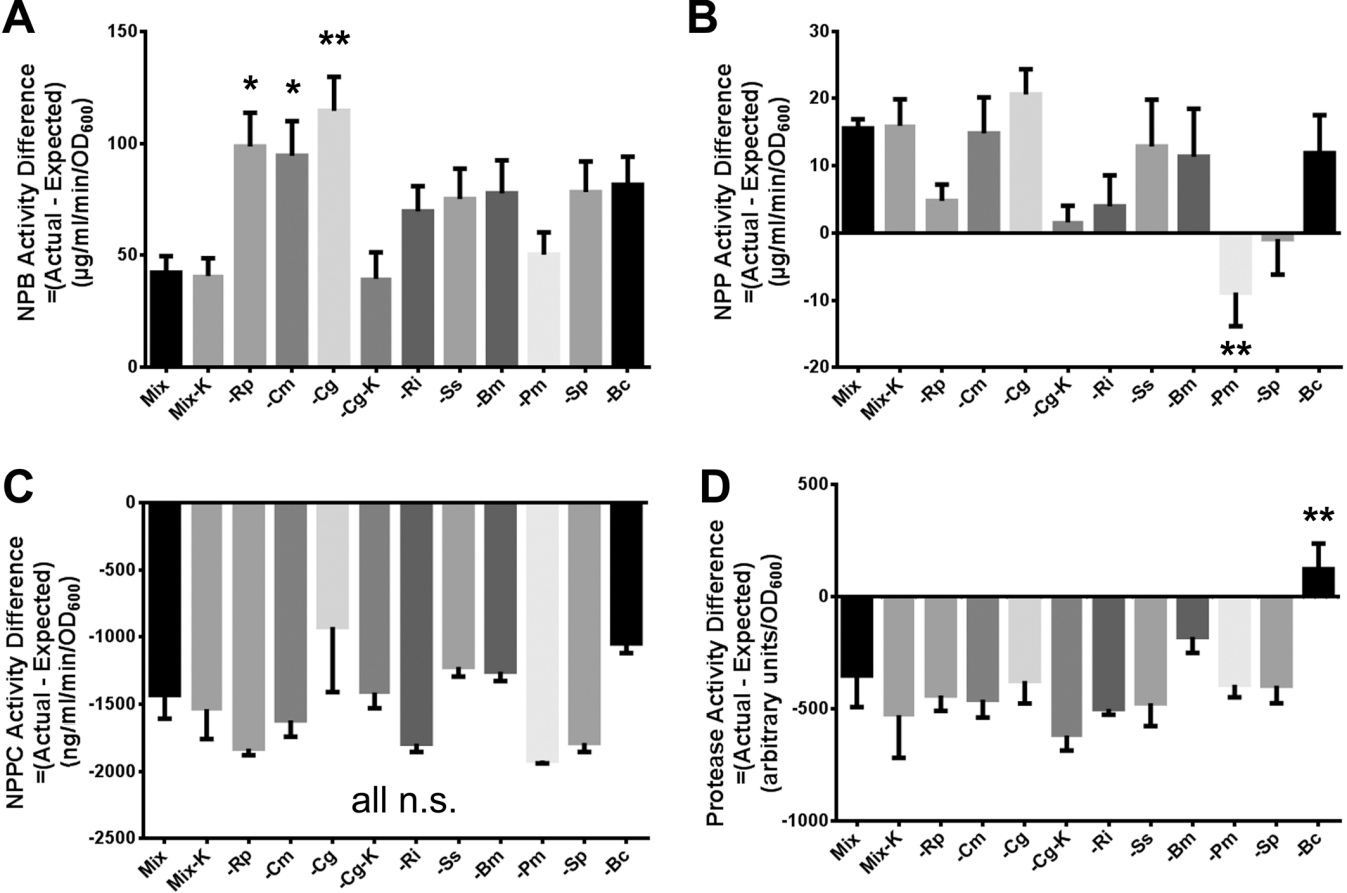
Effect of species absence on exoenzyme production from model communities using a comparison to predicted production based on single species cultures. Expected values for exoenzyme production were compared to measured value for (A) short-chain lipase activity, (B) long-chain lipase activity, (C) choline-specific phospholipase C activity, and (D) protease activity (from Fig 1). Error bars represent SEM. Samples all compared to ‘Mix’ (first bar) using ANOVA with Dunnett’s post-test. Statistical symbols: * = p < 0.05; ** = p < 0.01. Abbreviations: *Ralstonia pickettii* (Rp), *Cupriavidus metallidurans* (Cm), *Chryseobacterium gleum* (Cg), *Ralstonia insidiosa* (Ri), *Sphingomonas sanguinis* (Ss), *Burkholderia multivorans* (Bm), *Phyllobacterium myrsianacearum* (Pm), *Sphingomonas paucimobilis* (Sp), and *Burkholderia cepacia* (Bc). The Mix-K and–Cg-K denote predictions based on the final population proportion as measured in Table 2.

The only instance where secreted hydrolysis activity of the community was significantly altered by single species removal was for NPPC hydrolysis activity when *C*. *gleum* was excluded (Fig 1C). Three species were abundant producers of NPPC hydrolytic activity on their own (Fig 1C, right side of graph), therefore we examined whether *C*. *gleum* absence led to increased proportions of one or more of these high-NPPC hydrolysis activity producers (*S*. *sanguinis*, *B*. *multivorans*, and *B*. *cepacia*). To initially test this we took advantage of the fact that incubation at 37°C on Pseudomonas Isolation Agar allowed growth of only *Burkholderia multivorans* in this community. We used serial dilution-based colony counts to quantify *B*. *multivorans* in the full mix under these conditions, and the same conditions when *C*. *gleum* was absent, and observed no change in colony forming units between these conditions. We then used the differential plating strategy described in the Materials and Methods to assess the community composition in the presence or absence of *C*. *gleum*. We measured significant increases in the proportions of *Sphingomonas paucimobilis*, *Sphingomonas sanguinis*, and *Burkholderia cepacia* when *C*. *gleum* was removed (Table 2). However, the proportional rise in the population of these species was not numerically sufficient to explain the rise in NPPC hydrolysis activity directly (see Fig 3C, -Cg vs.–Cg-known). Basic ecological analyses of the community without *C*. *gleum*, based on the endpoint species proportions (Table 2), shows that the community has a Simpson’s Index of 0.46, a *ln*-based Shannon Index of 1.61, and a Equitability Index of 0.77.

Both *Burkholderia* species encode phospholipase C enzymes that are induced by choline breakdown products [50–52] and therefore typify the well-described regulation by metabolic intermediates [53, 54]. We could find no evidence in the literature that *C*. *gleum* synthesizes choline or phosphatidylcholine de novo, but limited studies on *C*. *gleum* make that conclusion uncertain. Therefore, a mechanism by which loss of *C*. *gleum* leads to increased NPPC hydrolysis activity remains elusive. It is possible that loss of *C*. *gleum* alters metabolism in the system leading to choline or choline-containing compound synthesis in some other members. We do know that the two Sphingomonads in the community can synthesize choline, phosphatidylcholine, and the choline-containing lipid sphinglomyelin de novo [55]. Both of the Sphingomonads increase in prevalence when *C*. *gleum* is absent (Table 2). While the strict numerical increases in *S*. *paucimobilis* and *S*. *sanguinis* populations are not sufficient to explain the increased hydrolysis activity (see the next subsection), these increases in population combined with alterations in metabolism could synergize to yield the increased activity. It is also possible that these species, or a response of the community as a whole leads to the production of esterases or phosphatases that also hydrolyze NPPC. Deletion of the phospholipase Cs in one or more members of this community would be a way of directly addressing this issue in the future.

### Evaluation of the utility of calculated expected values

To understand the factors regulating exoenzyme production, it is important to generate some simple expected values or numerical models that, minimally, account for differences in starting or final species composition within the community. This strategy is essentially a null hypothesis generator that takes a simple set of assumptions regarding population structure and enzyme production to predict the enzyme production levels in the mixed community. If all measured activities match these simple predictions (expected values), then community enzyme production is simply the sum of production from all members. This method is also a required prelude to establishing a computational model of such processes.

We used the activity of each species grown in monoculture to generate an “expected” activity for each given community composition using two methods, and compared these values to the actual activities measured from each community. The first method calculates enzyme activities based on the fractional input proportions of each community member [22], while the second calculates activity based on the fractional proportions measured after 72 h of static growth (greater detail in Materials and Methods section). For most communities, the expected enzyme activities are not equal to the measured activities, but the differences between actual and expected values are similar for most community compositions (Fig 3A and 3B). However, absence of *Ralstonia pickettii*, *Cupriavidus metallidurans*, or *Chryseobacterium gleum* each led to a statistically significant increase in the difference between actual and expected values compared to the complete community for NPB hydrolysis activity. Additionally, absence of *Phyllobacterium myrsianacearum* led to a statistically significant decrease in the difference between actual and expected values compared to the complete community in regard to NPP hydrolysis activity (Fig 3B).

In contrast to the lipase data, all measured NPPC activities for the species mixes were much lower than expected (Fig 3C). The direction and magnitude of this difference is due to the very high NPPC hydrolysis activities measured for single species cultures of *Sphingomonas sanguinis*, *Burkholderia multivorans*, and *Burkholderia cepacia* driving the calculated expected value higher.

When we subtract the expected protease activity from the measured activity, the full community and most of the multispecies communities with one species absent, have negative values (Fig 3D). This pattern is likely driven by high protease production by monocultures of *Sphingomonas sanguinis*, *Burkholderia multivorans*, and *Burkholderia cepacia*, the same three species with high NPPC hydrolysis activity when grown alone (compare the right sides of Fig 1C and Fig 1D). The only community with an (Actual–Expected) relationship that was significantly different from the full community was observed in the mix lacking *Burkholderia cepacia* (Fig 3D).

Based on our calculations of the relationship between actual and expected enzyme activity levels (Fig 3), neither the initial species proportions (most of the bars), nor the final species proportions (Mix-K and Cg-K bars), correlate well with measured enzyme activities. The exception is long-chain lipase activity, where the final species proportion in the–*C*. *gleum* community yields an expected value that is very close to the actual value (Fig 3B). However, it can be readily appreciated that the variations in expected values (Fig 3) do not directly correspond to measured activity levels (Fig 1, left side of each graph). Therefore, one can hypothesize regulatory processes that sculpt final enzymatic activity levels in the context of this community in this experimental environment.

One of the caveats to this work, or any study involving many bacterial species in a community, is that it is technically challenging to quantitatively know both the viable and functional populations of each organism throughout a particular experiment. Here, we have based our predicted enzyme activity on the sum of the final activity measures from each species divided by their proportion at the beginning of the experiment, or their proportion at the end of the experiment for two of the communities. Possibly due to the assumptions inherent in both approaches [as acknowledged in [22]], both sets of predicted values were equally far from the actual enzyme activity values (Fig 3).

## Conclusions

In conclusion, by quantifying exoenzyme production from a reconstructed model drinking-water microbial community and assessing the impact of individual species absence, we have described the functional robustness of lipase and protease production to species absence. This bacterial community, being diverse yet limited in species number, may be a tractable model community for identifying the mechanisms governing the regulation of community enzyme production in drinking water biofilms.

## Materials and Methods

### Strains, strain maintenance, and chemicals

*Ralstonia pickettii* 090890045–1, *Cupriavidus metallidurans* 101480065–2, *Chryseobacterium gleum* 113330055–2, *Ralstonia insidiosa* 130770013–1, *Sphingomonas sanguinis* 121850020–1, *Sphingomonas paucimobilis* 121220007–2, *Burkholderia multivorans* 122630001–1, *Burkholderia cepacia* 113330051–2, and *Phyllobacterium myrsianacearum* 113270001–2 were isolated from the International Space Station Potable Water Delivery System (except for *C*. *gleum*, isolated from the Russian SVO-ZV) and identified by the Microbiology Laboratory at the NASA Johnson Space Center (Houston, TX) [23]. The strain numbers listed after each species are for the strain database at the Johnson Space Center, and strains may be requested directly using these designation for all species described herein. All bacteria were stored in 20% glycerol stocks at -80°C and were streaked to R2A plates for recovery at 30°C.

All chemicals and media components were purchased from Fisher Scientific or Sigma Aldrich, except for ρ-nitrophenylphosphorylcholine (NPPC), which was purchased from Setareh Biotech.

### Community formation and preparation of supernatants for enzyme activity measurement

Colonies from fresh R2A plates were grown in R2B medium overnight and used to inoculated 500 μl of ½ strength R2B (diluted with distilled water) in 48-well tissue culture plates for an initial OD_600_ of 0.05 for each community member for a final community OD_600_ of ~0.45. The CFU/ml values at OD_600_ of 0.05 for each species with the exception of *C*. *gleum* were within 0.4 log10 units of each other, centered around 1E8 CFU/ml. This variance is close to the technical error level of our serial dilution plating (roughly 0.3 log10 in control counts). The CFU/ml for *C*. *gleum* was 0.4 log10 units below the mean of the other species. The community was allowed to form under static conditions (no shaking) at room temperature (20–23°C) for 72 h. This community contained both planktonic and biofilm components. Supernatants from the community were gathered by resuspending the contents of each well via vigorous pipetting and scraping of the well walls with the pipette tip. Since we were focused on products in the supernatant, bulk removal was assessed visually by crystal violet staining. Compared to the biomass removed, very little remained attached (<< 1%). At this point, an aliquot of the resuspended community was removed for CFU counts and differential plating as described below. This aliquot was vortexed vigorously and disruption of clumps assessed by light microscopy. While some clumps were present, they were not a substantial proportion of the population of cells.

To measure the effective OD_600_ from these wells, cells and debris were collected by centrifugation at 14,000 x g and supernatant removed for enzymatic testing. The resulting pellet was resuspended in R2B and the optical density at 600 nm was measured. All enzymatic activity assays were conducted on cell-free supernatants prepared as described above. Therefore the assays measure enzyme activity present at a single point in time (that of supernatant collection) and cells were not exposed to the high concentrations of colorimetric/fluorometric substrates, which would alter feedback regulation.

### Differential plating

Bacteria were grown on a variety of antibiotics at either 30°C or 37°C to establish susceptibilities that could be used for differential plating. These conditions and growth results are summarized in Table 1. To establish colony counts for the mixed biofilm community and the species removal leading to altered NPPC hydrolysis activity, mixtures were plated on LB^NS+Suc^ (LB with no salt and 5% sucrose) for total colony count, LB^Gm25^ (gentamicin 25 μg/ml), and LB^Tp100^ (trimethoprim 100 μg/ml). The LB^Tp100^ enabled determination of the two *Sphingomonas* species, as *S*. *sanguinis* is resistant to trimethoprim and *S*. *paucimobilis* is susceptible. LB^Tp100^ also enables separation of the two *Burkholderia* species, as *B*. *multivorans* is susceptible and *B*. *cepacia* is resistant to trimethoprim. Trimethoprim also allowed determination of *P*. *myrsianacearum* compared to all other white/beige colonies, as it grew well at 30°C on LB^Tp100^ plates, while it could not grow on gentamicin and also not at 37°C on any plate. *C*. *gleum* is a distinctive orange color, easily distinguished from either Sphingomonad. *Ralstonia insidiosa* was distinguished from *R*. *picketii* by the former’s resistance to gentamicin at 37°C. *C*. *metallidurans* and *R*. *insidiosa* are distinguished by the later having rough-edged colonies under these plate conditions.

### Lipase activity assays

Choline headgroup-specific phospholipase C hydrolysis was measured using the NPPC assay as previously described [56]. Briefly, 100 μl of sample was added to 100 μl of 50% glycerol, 50 mM NaCl, and 20 mM NPPC. Produced nitrophenol was measured using absorbance at 410 nm in a Biotek Synergy2 plate reader and converted to units of PLC (ng/ml/min/OD_600_) using the extinction coefficient of nitrophenol, 17,700/cm.

Measurements of short and long chain lipase activity were done as per the NPPC assay with ρ-nitrophenylbutyrate (NPB, short) or ρ-nitrophenylpalmitate (NPP, long) at 20 mM. For long chain lipase activity, the NPP was first dissolved in acetone before resuspension in the reaction buffer, which resulted in some NPP lost to insoluble material, but sufficient NPP in solution for rapid enzymatic determination. Hydrolysis was quantified as per the NPPC assay above. Activities for NPB and NPP hydrolysis were higher than for NPPC hydrolysis and are reported as μg/ml/min/OD_600_.

### Liposome formation and liposome lysis measurements

Liposomes were generated via the extrusion method based on the instructions and hardware specified by Avanti Polar Lipids. Liposomes were composed of 4:1 (w/w) phosphatidylcholine and cholesterol. Hydration was conducted in Dulbecco’s phosphate buffered saline (DPBS) in the presence of 500 nM rhodamine and the mixture was extruded through a 0.2 μm membrane using 11 unidirectional passes through the membrane. Liposomes were dialyzed in Slide-A-lyzer cassettes (20 kDa cutoff, Thermo Pierce) against DPBS to remove non-encapsulated rhodamine. These liposomes were stable for at least 48 h at 4°C and all assays were done within this timeframe.

Liposome lysis was measured according to the general method used to measure liposome stability [57]. Briefly, we added 20 μl of liposomes to 200 μl of supernatant and took 20 μl aliquots at 30 min intervals for two hours. Each aliquot was diluted with 80 μl DPBS and intact liposomes collected by centrifugation at 15,000 x g for 20 min. From the resulting supernatant, 50 μl was transferred to a white-walled 96-well microtiter dish and fluorescence measured with 530/25 nm excitation and 590/35 nm emission filters using a Biotek Synergy2 plate reader. Positive control for liposome lysis was based on dissolution of the liposomes with Triton X-100 detergent at a final concentration of 1%. This positive control was set as 100% lysis and lysis in other conditions reported as % lysis. The negative control for lysis was incubation of liposomes in heat-denatured supernatant. We also used, for comparison, supernatant from Pseudomonas aeruginosa PAO1 that secretes the protein hemolysis PlcH (prepped as above) and addition of purified PlcH at 1 ng/μl, prepared as described [58].

### Protease assay

Protease activity was measured using the Thermo Pierce Fluorescent Protease Assay Kit as modified here. The supernatants from the biofilm experiments were mixed with FITC-casein substrate in the reaction buffer described for the lipase activity (25% glycerol, 25 mM NaCl final concentrations). Fluorescence was read with 485/20 nm excitation and 528/20 nm emission filters using a Biotek Synergy2 plate reader immediately after assay start to measure baseline autofluorescence of the sample mixes and then after four hours incubation at room temperature.

### Statistical analysis and calculation of expected enzyme activities

For data in Figs 1–3, assays were run with three biological replicates in each of three independent experiments on different days. All data was included in the graphs and variance plotted as SEM. Statistical analysis was done using a one-way ANOVA with a Dunnett’s post-test where the full species mix was the comparator, except for Fig 2 where liposomes in heat-denatured supernatant was the comparator. For Table 2, a two-way ANOVA was used with a Sidak’s multiple comparisons test. These two tests were chosen as they are the most widely-used post-tests for comparison of multiple groups to a single control group from one-way (Dunnett’s) and two-way (Sidak’s) ANOVAs.

The expected values were generated using two methods, one based on the initial equal input of bacteria [22], and the other based on the measured proportions of species within the biofilm at 72 h. For each method, we divided the enzyme activity measured from a single species biofilm by its fractional starting proportion in the assay (1/9^th^ for the full mix, 1/8^th^ for each mix with a single species removed) or the proportion as measured at 72 h, and summed these corrected activities for a given mixture. Thus, each mixture has a different expected enzyme value based on the proportional contribution of the members.
